# Expression of the APOE4 allele influences the systemic environment to alter hippocampal phenotypes

**DOI:** 10.1002/alz.092692

**Published:** 2025-01-03

**Authors:** Sarah M Philippi, Ana Catarina Ferreira, Brittany M. Hemmer, Kailash BP, Manav Kapoor, Towfique Raj, Joseph M. Castellano

**Affiliations:** ^1^ Icahn School of Medicine at Mount Sinai, New York, NY USA

## Abstract

**Background:**

The apolipoprotein E (APOE) ε4 allele is the strongest genetic risk factor for Alzheimer’s disease (AD), increasing risk from 3‐12‐fold relative to the common ε3 allele. Seminal studies have revealed that age‐related changes in blood‐CNS communication regulate cognitive function. More recently, youth‐associated blood‐borne proteins revitalize the aged brain, improving hippocampal function and increasing adult neurogenesis and dendritic spine plasticity. Characterizing plasma proteomic changes across various systemic contexts may thus be critical for development of novel therapeutic strategies to combat AD. We hypothesized that the plasma proteome differs between *APOE4* and *APOE3* individuals, and these systemic changes account for differences in brain function according to *APOE* genotype.

**Method:**

Using an aptamer‐based platform to profile the plasma proteome from human *APOE4/4* and *APOE3/3* subjects, we identified allele‐specific changes in protein expression and canonical pathways linked to CNS functions and processes. Next, we examined the plasma proteome in *APOE* knock‐in mice expressing human *APOE3* or *APOE4* and evaluated those pathways that are conserved across species. To examine molecular processes within the brain regulated by opposing *APOE* alleles from the systemic environment, we characterized transcriptomic changes in the hippocampi of mice in which blood is shared between *APOE4* and *APOE3* mice by parabiosis relative to isogenic control pairs.

**Result:**

In both mouse and human subjects, expression of *APOE4* is associated with perturbations of plasma proteins related to extracellular matrix (ECM) and inflammation pathways relative to expression of *APOE3*. Several pathways shown to be associated with *APOE3* expression in the hippocampus and disrupted in *APOE4*‐KI hippocampus were found to be restored in *APOE4*‐KI mice exposed to *APOE3* blood via parabiosis. Specific deleterious phenotypes were identified in the hippocampi of *APOE3* mice exposed to *APOE4* blood, suggesting that *APOE4*‐associated brain phenotypes are, in part, mediated by how *APOE4* alters the systemic compartment.

**Conclusion:**

We find that *APOE* alleles differentially alter the systemic environment to confer changes in brain phenotypes. Ongoing experiments focus on investigating the protective impact of the *APOE*3 systemic environment on molecular processes within the *APOE4* hippocampus, which may ultimately inform our understanding of increased AD risk in *APOE4* subjects.

